# Editorial: Flavonoids and Cardiovascular Metabolism

**DOI:** 10.3389/fnut.2022.939798

**Published:** 2022-06-22

**Authors:** Qiao Zhang, Shi-Jun Yue

**Affiliations:** ^1^Key Laboratory of Shaanxi Administration of Traditional Chinese Medicine for TCM Compatibility, State Key Laboratory of Research & Development of Characteristic Qin Medicine Resources (Cultivation), Shaanxi University of Chinese Medicine, Xi'an, China; ^2^College of Pharmacy, Shaanxi University of Chinese Medicine, Xi'an, China

**Keywords:** flavonoid, cardiovascular diseases, inflammation, oxidative stress, gut microbiota

Flavonoids are one of the most abundant and bioactive components in herbs, plants and fruits, and are widely used in various nutritional products, cosmetics, and medicines. Flavonoids have a wide range of pharmacological effects, such as inhibiting the secretion of inflammatory factors and reducing oxidative stress. Cardiovascular disease is still the main reason for disease-related death worldwide, causing substantial economic, and social burdens. Massive evidence has demonstrated that inflammation and oxidative stress are common features in various cardiovascular diseases. Given the prophylactic and therapeutical benefits flavonoids on cardiovascular disease, more research is necessary to clarify the exact role and mechanism of flavonoids on cardiovascular diseases, especially from the perspective of metabolism. This collection of articles consists of three review article and a research paper that explore the impact of natural flavonoids on cardiovascular and metabolic diseases from different standpoints.

Apigenin (4′,5,7-trihydroxyflflavone) is a star molecule from nature. Xu et al. summarize the underlying mechanisms of apigenin-induced biological response in multiple cardiometabolic diseases, including obesity, diabetes, hypertension, and cardiovascular diseases. Specifically, apigenin alleviates obesity and its complications via the inhibition of appetite, glucose signaling pathway and lipid metabolism, but also the regulation of gut microbiota, the reinforcement of mitochondrial function and the alleviation of inflammation and oxidative stress. The apigenin-mediated regulation of blood glucose homeostasis is achieved mainly through regulating the key enzymes and improving oxidative stress as well as inflammation. Apigenin decreases blood pressure mainly through improving nitric oxide bioactivity and oxidative stress, regulating apoptosis-related mitochondrial genes, and promoting vascular endothelium-dependent vasodilation. Likewise, apigenin ameliorates cardiovascular diseases via reduction of oxidative stress and mitochondria-dependent apoptosis in vascular endothelial cells as well as regulation of glycolipid metabolism in cardiomyocytes. Nevertheless, the authors emphasize that more attention shall be paid to the contrasting roles of apigenin in different types of cells. Moreover, design better clinical trials to confirm the preventive/therapeutic usefulness of apigenin appear essential and important.

*Ginkgo biloba*, known as the “living fossil,” has a long history of medicinal use. More than 110 different kinds of *G. biloba* flavonoids have been uncovered, including flavones, flavonols, biflavonoids, catechins, and their glycosides. Tao et al. focus on the metabolic regulatory processes and gene regulation of cellular metabolism in cardiovascular diseases of *G. biloba* flavonoids. The metabolism of flavonoids is mainly mediated by hepatic UGT1A9 enzyme, while the glucuronidation and hydrolysis of flavonoids into aglycones are accomplished in the small intestine under the fermentation of gut microbiota. At the same time, *G. biloba* flavonoids can modulate the structure and function of gut microbiota to remotely treat cardiovascular diseases. So, understanding the pharmacokinetic and metabolic information of *G. biloba* flavonoids could shed light on the cardioprotective mechanism of *G. biloba* flavonoids. Till now, some star molecules including quercetin, kaempferol, apigenin, and luteolin of *G. biloba* have been proven to protect myocardial ischemia-reperfusion injury, protect endothelial cell damage, and prevent coronary atherosclerosis, but other flavonoids especially biflavonoids of *G. biloba* are less studied and their mechanism of action remain to be elucidated.

Li et al. discuss the potential effects and mechanism of natural flavonoids from fruits in the treatment of atherosclerosis. Fruit flavonoids such as apigenin, quercetin, kaempferol, rutin, naringin, and dihydromyricetin are “pleiotropic” compounds and have therapeutic effects on atherosclerosis by protecting endothelial cell dysfunction, inhibiting foam cell formation, regulating lipid metabolism, and anti-inflammation. The druggability of natural flavonoids, however, remains an obstacle to new drug research and development. The design of novel delivery systems such as nanomaterials for flavonoid therapy is an important direction in future research. Such knowledge could pave the way for the development of dietary interventions/new drugs for prevention and treatment of cardiometabolic diseases.

Recently, fruit vinegars such as coconut vinegar have been consumed in increasing amounts for their promising therapeutic properties, which could be related to a variety of polyphenolic compounds that they contain. Research by Malakul et al. elucidates the protective effects of daily supplementation of new coconut vinegar prepared by themselves in high-cholesterol diet fed rats. The authors identify coconut vinegar could reduce the accumulation of both hepatic cholesterol and hepatic triglyceride, and the hepatic 4-hydroxynonenal lipid peroxidation. Protection against vascular oxidative stress demonstrates a key biological function of coconut vinegar, which may be ascribed to flavonoids wherein. More evidence is needed before coconut vinegar could offer an alternative to the statin intolerance dyslipidemia patients, but also to high-risk individuals with hepatic steatosis and cardiovascular diseases. Undoubtedly, developing health-related medicine and food homology products containing natural flavonoids will be an inevitable trend.

To summarize, this collection of review and research articles gives us a comprehensive understanding of natural flavonoids in fruits, *G. biloba*, coconut vinegars and their positive impact on in cardiometabolic diseases. Flavonoids can interact with different cellular targets and intercept multiple pathways involved in cardiometabolic diseases. Interest in the interaction of gut microbiota with flavonoids has also increased in recent years. Gut-heart axis in the protective effects of flavonoids on cardiovascular diseases has been proposed. However, the efficacy of flavonoids in patients with cardiometabolic diseases and their ability to synergize with conventional drugs need to be explored further.

## Author Contributions

S-JY and QZ wrote the editorial. Both authors contributed to the article and approved the submitted version.

## Conflict of Interest

The authors declare that the research was conducted in the absence of any commercial or financial relationships that could be construed as a potential conflict of interest.

## Publisher's Note

All claims expressed in this article are solely those of the authors and do not necessarily represent those of their affiliated organizations, or those of the publisher, the editors and the reviewers. Any product that may be evaluated in this article, or claim that may be made by its manufacturer, is not guaranteed or endorsed by the publisher.

